# Turning over New Leaves

**Published:** 1890-06-28

**Authors:** 


### Turning Over New Leaves.

**On the State of Mind called Attention.**

Attention is that state of the mind in which an individual concentrates all his thoughts for the moment on one particular subject or object. Without a certain amount of attention we cannot be conscious of the impressions which impinge upon our sense organs. For instance, we may be unaware of the clock ticking on our mantel shelf until something occurs to direct our attention to it, then we immediately become conscious of a corresponding auditory impression. All the thoughts of our minds only exist for us in so far as we *attend* to them. Attention, therefore, is one of the most important faculties, or rather attitudes, of the mind, and is worthy of the closeststudy, far more indeed than has hitherto been given to it.

To the parent or teacher it has the highest interest for educational reasons, since it is only, or chiefly, during the early periods of life that the control of the attention, which gives so important a cast to the after career of the individual, is inculcated into the mind. Lack of perseverance is to a large extent a want of power to control the attention. And how often do we hear parents deplore this failing in their offspring; yet, probably, they are themselves to blame for having omitted to cultivate the quality in the golden hey-day of youth. This faculty differs very widely in different individuals, and speaking generally inattention is a prominent feature in weak-minded persons; and conversely a power to direct the attention, in spite of many diverse attractions, is an evidence of strength of mind.

There are, however, two forms of Attention which are more or less distinct from each other; instinctive, or natural attention, and voluntary, or artificial attention. The instinctive, or spontaneous form is that which exists naturally in a greater or less degree, and is born with nearly every living creature. It is always aroused by some *emotional* state, being attracted by whatsoever pleases and repelled by whatsoever pains the individual. The kind of spontaneous attention evinced by any person reveals his character and tastes. Thus, the attention of a frivolous woman is taken up with bonnets and the gossip of her neighbours. The doctor lends his attention to a scientific question in which a dock labourer would only see unmeaning nonsense. The inborn nurse lends hers to the "hydrocephalic cry" of an infant in which an unsympathetic woman only hears a disagreeable noise. It is by a close and careful study—parents, note this well!—of the direction in which a child's spontaneous or natural attention is directed that you are enabled subsequently to set them to the occupation in which he or she will be happiest, to which he is most suited, and in which, consequently, he will achieve the greatest success. There are thousands and thousands of lives wasted through a want of appreciation of this fact by the guardians of our youth. How common it is to see "the right man in the wrong place," dragging on his weary existence *because he cannot concentrate his attention* on his daily work, which is altogether unsuited to his tastes. Indeed, it may be taken as almost certain that a large number of the social failures who throng our workhouses are thus produced. There is assuredly, in this great universe, work of some kind appropriate to each individual, however insignificant; some kind of work suitable to attract and retain his attention; and it is the duty of the parents and teachers to ascertain in early life *the bent of the Attention*, so as to mould the after life accordingly. It is this problem which is so sadly neglected in the Board Schools and other educational establishments of the present day where the training of each and all is set at a dead level uniformity.

Turning next to voluntary or artificial, or, as it is sometimes called, Active Attention, here again the question of education comes in even with greater force; for this kind is entirely the product of art, habit, and training. It is essentially dependent on the will, which sets its direction. The power of voluntary Attention is cultivated by rendering attractive to the individual, by artifice, what is not so by nature; by giving an artificial interest to things which have not a natural one. This may be done in many ways. Curiosity, novelty, hope of reward, pleasant collateral surroundings, emulation, ambition, the organic needs of daily existence, are some of the means by which voluntary attention may be secured and the habit cultivated. An initial, innate, power of will is doubtless of much importance for its culture, but a great deal may be done by careful and appropriate training for its development.

There is a little book on the Psychology of Attention, which has recently appeared in English which is well worthy of perusal.* The third part, treating of morbid condition of Attention, is undoubtedly the most interesting. Professor Ribot considers that the power of artificial attention originates in voluntary control of the muscles. Attention is directed along some particular path by voluntary active *inhibition* (the result of effort) or restraint of muscular action in all other directions. "In all cases of attention there must necessarily be a play of muscular elements (real or nascent movements), upon which the power of inhibition acts." This is the author's standpoint for normal Attention.

He classifies abnormal states into (1) Hyper, or over Attention, (2) Atrophy of Attention, and (3) Congenital deficiency of Attention.

Hypertrophy of Attention, or, as we should prefer to call it, Hyper-attention is "an *absolute* predominence of one state or group of states" to the complete exclusion of all others. As examples, hypochondriasis, fixed idea, and ecstasy may be mentioned. *Fixed idea* is a condition of mind in which the individual's thoughts continually and involuntarily revert to one notion. As a matter of fact in every sound human being there is always a *dominant* idea that regulates his conduct. "The metamorphosis of attention," says Professor Ribot, "into a fixed idea is much more clearly seen in great men," and he quotes Alfred de Vigny's question and answer, "What is a great life?" "A thought of our youth, realised in mature age."

In keeping with the methodical character of the book, a feature which everywhere pervades its pages, the author classifies fixed ideas into three great categories, (1) Simple fixed ideas of a purely intellectual nature; (2) Fixed ideas accompanied by emotions, such as terror; (3) Fixed ideas of an impulsive form, known as irresistible tendencies, that manifest themselves in violent or criminal acts. If a moment's reflection be devoted to this third variety of disordered Attention, the transcendent importance of training the Attention aright in early life becomes again apparent from another point of view.

Atrophy of, or abnormally deficient, Attention, may be due (1) to an abnormal rapidity and exuberance of ideas, so that all is disorder and no particular state of consciousness lasts even for a moment, as in cases of delirium or acute mania. Or (2) it may be due, and this is more frequent, to an absence or diminution of the power of inhibition (restraint). "Numerous instances of this are met with in hysterical patients, in persons suffering from irritable weakness, in convalescents, in apathetic and insensible individuals, intoxication, and extreme states of bodily or mental fatigue." Deficient Attention and motor weakness go side by side; and this is one of the facts on which the author relies to show the motory origin of the faculty.

* "The Psychology of Attention." By Th. Ribot, Professor of Comparative and Experimental Psychology at the College de France. Authorised translation, pp. 120. The Open Court Publishing Company, Chicago. (Messrs. Longman's and Co., London.)

